# Unveiling pediatric secondary hemophagocytic lymphohistiocytosis: a comprehensive analysis of etiology, diagnosis, and treatment

**DOI:** 10.3389/fimmu.2026.1753930

**Published:** 2026-04-24

**Authors:** Ping Liu, Min Wang, Chuanwei Ban, Yumeng Ma, Juan Wang, Xin Lv

**Affiliations:** 1 Clinical Laboratory, Children’s Hospital Affiliated to Shandong University, Jinan, China; 2 Clinical Laboratory, Jinan Children’s Hospital, Jinan, China

**Keywords:** clinical manifestation, hemophagocytic lymphohistiocytosis, laboratory features, pediatric, treatment

## Abstract

**Objective:**

To describe the etiological spectrum, treatment approaches, clinical and laboratory characteristics in pediatric secondary hemophagocytic lymphohistiocytosis (sHLH) to improve awareness of this severe illness and summarize evolving management strategies.

**Methods:**

A retrospective analysis was conducted on 110 children initially diagnosed with sHLH at our hospital between January 1, 2018, and June 30, 2025.

**Results:**

Among 110 sHLH patients, the median age at diagnosis was 2.67 years (1.17, 5.96), and 52.7% were under 3 years old. Infection-associated HLH accounted for 78.2%, with Epstein-Barr virus (EBV) as the most common trigger (57.3%). The remaining cases were attributed to rheumatic or malignant diseases. The main clinical manifestations included fever (99.1%), lymphadenopathy (83.6%), splenomegaly (77.3%), and hepatomegaly (66.3%). Respiratory system involvement was observed in over half of the patients, while central nervous system involvement (CNSI) and multiple organ dysfunction syndrome (MODS) occurred in 22.7% and 12.8% of cases, respectively. Characteristic laboratory abnormalities were highly prevalent, including pancytopenia (especially thrombocytopenia), hyperferritinemia, hypofibrinogenemia, and elevated soluble interleukin-2 receptor (sCD25). Most patients showed varying degrees of hepatic dysfunction, mainly with elevated enzymes (LDH, AST, ALT, HBDH). Characteristic immunological abnormalities included a decreased NK cell proportion (75.5%) and a reduced CD4^+^/CD8^+^ ratio (59.1%). Regarding therapy, 44.5% of patients received the HLH-94/04 chemotherapy protocol, among these, 75.5% had EBV infection, with a chemotherapy remission rate of 91.9%. The overall in-hospital mortality was 13.6%, with MODS accounting for 73.3% of fatalities.

**Conclusions:**

Pediatric sHLH is a severe, multisystem inflammatory disorder that predominantly affects infants and young children, with EBV infection as the primary etiological trigger. In addition to the classic HLH-2004 criteria, abnormal liver function indicators, imbalanced lymphocyte subsets and respiratory system involvement were frequent salient features, suggesting their potential utility as auxiliary diagnostic indicators. Furthermore, our findings further emphasize the importance of etiology-based individualized treatment.

## Introduction

Hemophagocytic syndrome (HPS), also known as hemophagocytic lymphohistiocytosis (HLH), represents a critical inflammatory disorder resulting from the hyperactivation of the immune system. The fundamental pathophysiology involves functional impairments in cytotoxic T cells and natural killer (NK) cells, which result in uncontrolled immune reactions and a significant release of inflammatory cytokines, thereby inducing a systemic hyper-inflammatory response (1, 2). Clinically, this syndrome manifests as persistent fever, hepatosplenomegaly, pancytopenia, along with elevated levels of ferritin and triglycerides. Pathologically, the predominant feature is the excessive proliferation and phagocytic activity of histiocytes or macrophages within the reticuloendothelial system, including the bone marrow, liver, spleen, and lymph nodes (3, 4).

Based on the presence of underlying genetic defects, HLH is classified into primary HLH and secondary HLH (sHLH) (2). Primary HLH, also known as familial HLH (FHL), is a hereditary disorder caused by genetic mutations and is more common in children (5, 6). Secondary HLH can be triggered by various factors such as infections, malignancies, and autoimmune diseases (7), which are termed infection-associated HLH (IAHLH), malignancy- associated HLH (MAHLH), and autoimmune disease-associated HLH, respectively (8). Common pathogens associated with IAHLH include viruses, bacteria, mycoplasmas, parasites, and fungi, etc. Among the pediatric population, Epstein-Barr virus (EBV) infection is the most common trigger and is often associated with severe illness (7, 9). Interestingly, macrophage activation syndrome (MAS) is a crucial subtype of sHLH associated with rheumatic diseases (8). Systemic juvenile idiopathic arthritis (sJIA) is the most predominant trigger, with approximately 10% of sJIA patients potentially developing concurrent MAS. Additionally, autoimmune diseases such as systemic lupus erythematosus (SLE) and Kawasaki disease (KD) may also induce MAS (10, 11).

Although the international HLH-2004 diagnostic and therapeutic protocol has improved the management of pediatric HLH (12), the nonspecific clinical manifestations and varying disease characteristics across age groups often lead to initial misdiagnosis as severe infection, sepsis, or hematologic malignancies, resulting in delayed intervention (13). Furthermore, HLH carries a relatively high mortality rate due to complications such as visceral hemorrhage, opportunistic infections, or multi-organ failure, underscoring the need for further research to optimize patient-centered outcomes (3, 14).Currently, comprehensive clinical studies on pediatric sHLH with substantial sample sizes, particularly those analyzing etiological distribution, phenotypic variability, and heterogeneity in laboratory parameters, are still relatively limited. To address this gap, we conducted a retrospective analysis of clinical data from 110 pediatric patients with sHLH admitted to our institution. This study aims to elucidate the clinical features, etiological spectrum, laboratory findings, treatment strategies, and outcomes of sHLH, thereby enhancing pediatricians’ awareness, and providing an evidence-based foundation for early diagnosis, rational therapeutic intervention, and improved prognosis in affected children.

## Materials and methods

### Study design and patients

This retrospective study collected the basic clinical data from pediatric patients first diagnosed with sHLH at the Children’s Hospital Affiliated to Shandong University between January 1, 2018, and June 30, 2025.The inclusion criteria complied with the HLH diagnostic criteria established by the Histiocyte Society (12), as follows: (1)fever; (2) splenomegaly; (3) cytopenias affecting two or more blood cell lineages: hemoglobin <90g/L (in infants <4 weeks: hemoglobin <100 g/L), platelets <100×10^9^/L, and neutrophils <1.0×10^9^/L; (4) hypertriglyceridemia (≥3.0mmol/L) and/or hypofibrinogenemia (≤1.5 g/L); (5) hemophagocytosis observed in the bone marrow, spleen, liver, and lymph nodes; (6) low or absent NK cell activity; (7) ferritin levels ≥500μg/L; (8) soluble interleukin-2 receptor levels (sCD25) ≥2400U/ml. Enrolled pediatric patients were required to meet at least five of the eight criteria. The age range was 0–14 years to cover all pediatric stages.

During the study period, 126 patients were diagnosed with HLH. Among them, 115 patients underwent HLH-related genetic testing. Five cases were genetically confirmed as primary HLH (2 with PRF1 mutations, 1 with STXBP2 mutation, 1 with LYST mutation, and 1 with GATA2 mutation). The remaining 110 tested patients without definitive pathogenic mutations associated with primary HLH and were classified as sHLH based on clinical features. An additional 11 pediatric cases were excluded due to either incomplete clinical data and lack of HLH-related genetic analysis. Consequently, 110 sHLH cases with clear clinical diagnoses were included as the final study subjects.

This study received approval from the Ethical Committee of the Children’s Hospital Affiliated to Shandong University (SDFE-IRB/P-2024087) in accordance with the Declaration of Helsinki, and laboratory data were anonymized prior to analysis.

### Research methods

Clinical data were carefully reviewed from the electronic medical record system, including initial consultation records, hospitalization progress notes, examination reports, treatment plans, and medication records, etc. The included research materials mainly consist of the following aspects: (1) baseline characteristics, including sex, age at diagnosis, and department distribution at the time of consultation; (2) etiological distribution, including types of infectious pathogens, information related to autoimmune diseases, and tumor-associated factors; (3) clinical manifestations, including fever, presence of hepatosplenomegaly and lymphadenopathy (determined by palpation and imaging), symptoms involving the respiratory, digestive, and urinary systems, neurological damage, and serous cavity effusion; (4) laboratory examinations, such as complete blood count, coagulation function, blood chemistry, lymphocyte subsets, cytokines, C-reactive protein (CRP), and bone marrow hemophagocytosis; (5) treatment and in-hospital outcome. All data were obtained from the patients’ first visit to our hospital.

### Statistical analyses

Statistical analysis was conducted using SPSS software (version 23.0; SPSS, Chicago, IL). Continuous data are presented as medians with interquartile ranges [M (Q1, Q3)], and categorical data are presented as absolute numbers and percentages.

## Result

### Baseline characteristics of patients with sHLH

Among the 110 **s**HLH patients, 63 (57.3%) were male and 47 (42.7%) were female, and the median age at diagnosis was 2.67 years (1.17, 5.96). A total of 58 patients (52.7%) were under 3 years old, 31 (28.2%) were between 3 and 6 years, and 21 (19.1%) were over 6 years old. In this retrospective analysis, sHLH patients were initially diagnosed and confirmed across 12 departments, including Hematology, Critical Care Medicine, Rheumatology and Immunology, Infectious Diseases, Cardiovascular Medicine, and Neonatology, among others. Twenty-one patients were directly admitted to the Pediatric Intensive Care Unit (PICU), and 8 cases were transferred from other departments. The median length of initial hospitalization was 15.0 (10.0, 18.0) days, and median diagnostic time was 3.0 (2.0, 6.0) days.

### Etiological analysis of patients with sHLH

**Figure 1:**
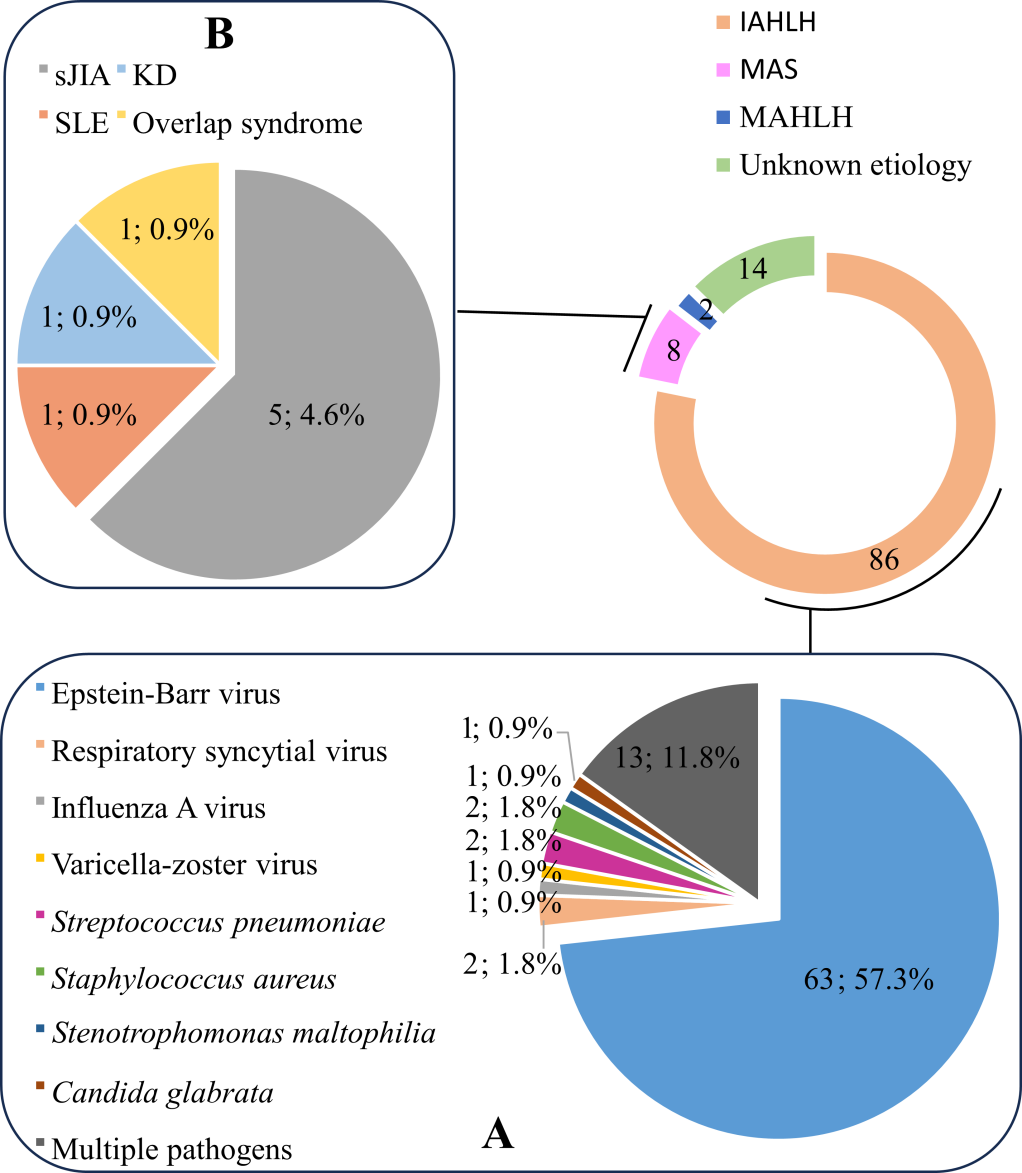
Etiological distribution of sHLH in 110 children. **(A)** IAHLH, Infection-associated hemophagocytic lymphohistiocytosis. **(B)** MAS, Macrophage activation syndrome.

In our cohort, 86 cases (78.2%) were classified as IAHLH. As detailed in **Figure 1A**, EBV was the predominant trigger, accounting for 57.3% of all cases. Other detected pathogens included other viruses, bacteria, and fungi. Notably, 11.8% of IAHLH cases involved co-infections with multiple pathogens. The remaining 24 patients (21.8%) constituted the non-infection-associated HLH group, including: (i) 8 patients (7.3%) with MAS (**Figure 1B**); (ii) 2 patients (1.8%) with MAHLH, specifically one case of Anaplastic Large Cell Lymphoma and one case of Neuroblastoma, both directly triggered by the underlying malignancy; and (iii) 14 patients (12.7%) in whom no specific trigger could be identified, categorized as HLH of unknown etiology.

### Clinical manifestations of patients with sHLH

The clinical manifestations in this cohort were diverse. The most common were fever, lymphadenopathy, splenomegaly, and hepatomegaly. Over half of the patients exhibited respiratory system involvement, including rhinorrhea, cough, dyspnea, and wheezing. The main radiographic findings were pneumonia and pleural effusion. Gastrointestinal manifestations were also prevalent in this cohort, encompassing vomiting, abdominal distension, diarrhea, and gastrointestinal hemorrhage, with ascites observed in 55.5% of patients. Additionally, central nervous system involvement (CNSI) was noted in 22.7% of patients, manifesting as convulsions, dizziness, drowsiness, coma, elevated cerebrospinal fluid protein and cell counts, and/or abnormal electroencephalography or cranial imaging. However, skin and urinary system symptoms were relatively rare. Among them, children with MAS often had rashes (87.5%, 7/8), typically presenting as an erythematous maculopapular rash, which was considered part of the systemic inflammatory presentation of MAS, rather than being caused by an infection. Further, a small group of patients presented with progressive multiple organ dysfunction syndrome (MODS) requiring intensive care. A detailed breakdown of all clinical manifestations is provided in **Table 1**.

**Table 1:** Clinical manifestations in the 110 children with sHLH.

Clinical manifestations	Case (n)	Proportion (%)
Fever	109	99.1
Lymphadenopathy	92	83.6
Splenomegaly	85	77.3
Hepatomegaly	73	66.4
Respiratory manifestations		
Pneumonia	64	58.2
Bronchitis	12	10.9
Respiratory failure	24	21.8
Pulmonary hemorrhage	2	1.8
Skin manifestations		
Rash	28	25.5
Bleeding point	10	9.1
Digestive system manifestations		
Nonprojectile vomiting	17	15.5
Abdominal pain	11	10.0
Diarrhea	10	9.1
Gastrointestinal bleeding	6	5.5
Abdominal distension	4	3.6
Urinary system manifestations		
Oliguria	3	2.7
Hematuria	2	1.8
Serous cavity effusion		
Ascites	61	55.5
Pleural effusion	40	36.4
Pericardial effusion	17	15.5
Other key indicators		
CNSI	25	22.7
MODS	14	12.8
Joint pain	8	7.3

CNSI, Central nervous system involvement; MODS, Multiple organ dysfunction syndrome.

### Laboratory features of patients with sHLH

Laboratory tests revealed bicytopenia or pancytopenia in nearly half of the patients, with thrombocytopenia and neutropenia being particularly common. Notably, only 23 children had a hemoglobin level below 90g/L.

Analyzing biochemical parameters is crucial for gathering credible evidence regarding the development of sHLH. Liver function abnormalities were prominent, with over 80% of cases exhibited abnormal increases in aspartate transaminase (AST) and lactate dehydrogenase (LDH). Fasting triglyceride levels were raised in more than two-thirds of the patients (68.2%, 75/110), although only a small fraction surpassed 3.0mmol/L. Hyperferritinemia (>500μg/L), a key indicator of HLH, was observed in 89.1% of cases. Additionally, hypoalbuminemia and hyperbilirubinemia were found in approximately one-third of patients.

Coagulopathy, evidenced by hypofibrinogenemia and elevated D-dimer, was present in 50.0% and 60.0% of patients, respectively. Lymphocyte subset analysis revealed that the most frequent abnormality was a decreased NK cell (CD16^+^CD56^+^) rate, followed by an increased CD3^+^ cell rate and a reduced CD4^+^/CD8^+^ ratio, the latter was accompanied by an elevated CD8^+^ cell rate (as detailed in **Table 2**). Among the 82 patients who underwent both sCD25 and NK cell activity testing, the diagnostic coincidence rate of elevated sCD25 was as high as 96.3%, and 59.8% had decreased NK cell activity. For cytokine detection, interleukin (IL)-10 elevation was the most common. Moreover, Bone marrow hemophagocytosis, an important pathological feature of HLH, was also frequently detected in the cohort. Laboratory parameters of sHLH are summarized in **Table 2**.

**Table 2:** A summary of laboratory parameters in the 110 children with sHLH.

Parameters	Case (n)	Percentage (%)
Complete blood counts		
Bicytopenia or tricytopenia	51	46.4
Hemoglobin < 90 g/L	23	20.9
NEUT < 1.0×10^9^/L	48	43.6
PLT < 100×10^9^/L	69	62.7
Biochemical features		
Increased ALT (>2 ULN)	78	70.9
Increased AST (>2 ULN)	91	82.7
Decreased albumin (<30 g/L)	34	30.9
Increased bilirubin (>2 ULN)	27	24.6
Increased LDH (>500 U/L)	93	84.6
Increased HBDH (>500 U/L)	72	65.5
Triglycerides (>3.0 mmol/L)	32	29.1
Ferritin (>500 μg/L)	98	89.1
Coagulopathy features		
APTT > 38s	35	31.8
PT > 14s	43	39.1
FIB < 1.5 g/L	55	50.0
D-Dimer > 5 mg/L	66	60.0
Immunological features		
Increased CD3^+^ cell rate	76	69.1
Increased CD8^+^ cell rate	67	60.9
Decreased CD4^+^ cell rate	52	47.3
Decreased CD4^+^/CD8^+^ ratio	65	59.1
Decreased CD19^+^ cell rate	51	46.4
Decreased CD16^+^CD56^+^ cell rate	83	75.5
Decreased NK cell activity ^a^	49	59.8
Increased sCD25 ^a^	79	96.3
Cytokine ^b^(>3ULN)		
IL-6 (pg/mL)	28	29.5
IL-10 (pg/mL)	79	83.2
IFN-γ (pg/mL)	45	47.4
Increased CRP (mg/L)	65	59.1
Hemophagocytosis in bone marrow ^c^	70	66.0

a. Only 82 cases underwent sCD25 and NK cell activity testing.

b. Only 95 cases underwent cytokines testing.

c. Only 106 cases underwent bone marrow testing.

ULN, Upper limit of normal; NEUT, Neutrophil Count, PLT, Platelet; ALT, Alanine transaminase; AST, Aspartate transaminase; LDH, Lactate dehydrogenase; HBDH, Hydroxybutyrate dehydrogenase; APTT, Activated partial thromboplastin time; PT, Prothrombin time; FIB, Fibrinogen; sCD25, Soluble interleukin-2 receptor; NK, Natural killer; IL, Interleukin; IFN-γ, Interferon-gamma; CRP, C-reactive protein.

### Treatment and outcome of patients with sHLH

In this study, almost all sHLH patients (90.9%, 100/110) received glucocorticoid treatment (dexamethasone or methylprednisolone), with intravenous immunoglobulins (IVIG; 81.8%, 90/110) and cyclosporine (48.2%, 53/110) serving as the main concomitant immunosuppressive agents. Most patients received antibiotics (87.3%, 96/110), and antiviral medications (72.7%, 80/110), while antifungal therapy was less common (1.8%, 2/110). Additionally, transfusion therapy and mechanical ventilation were used in 67.3% and 21.8% of patients, respectively.

**Figure 2:**
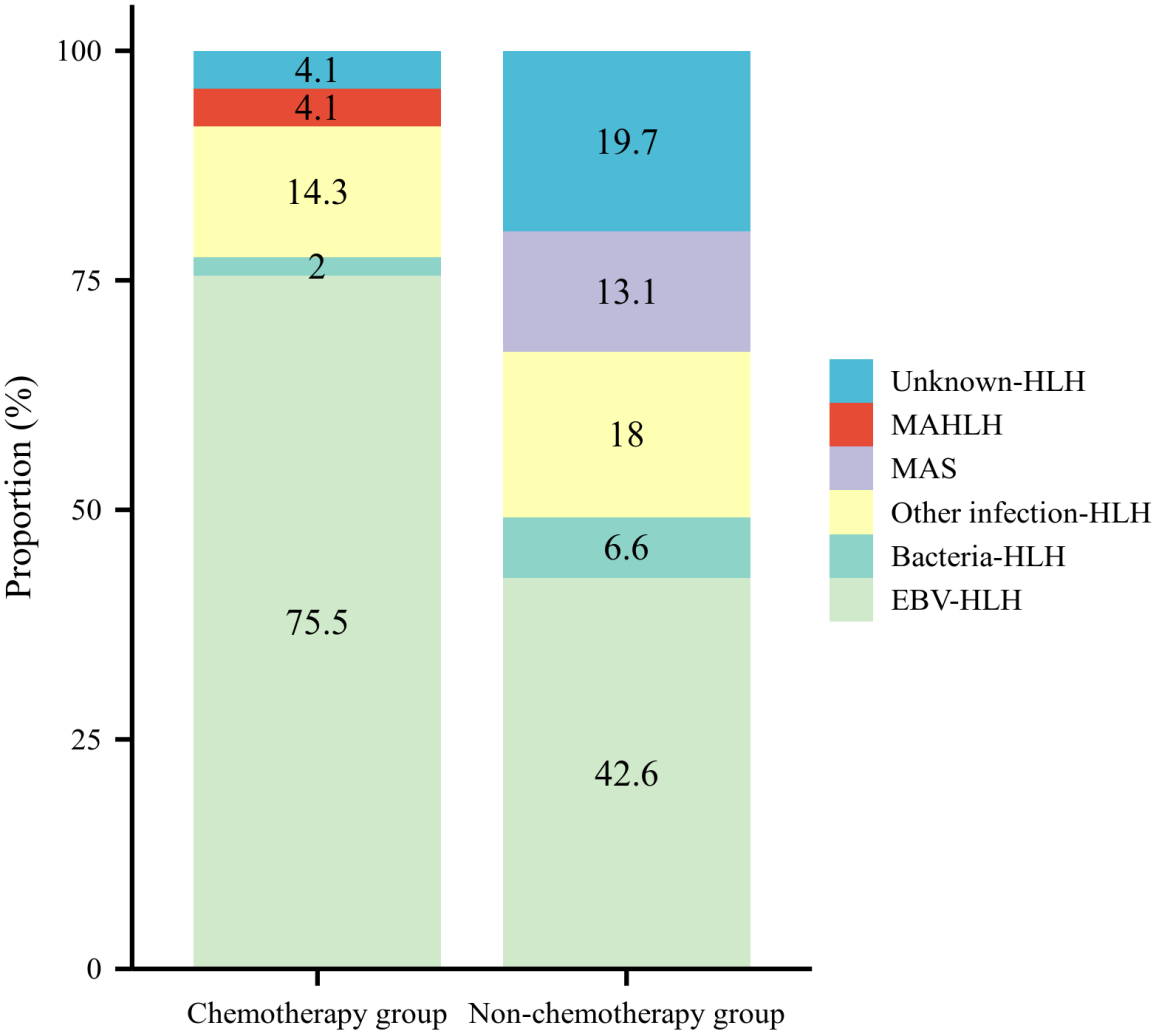
The therapeutic approaches of different subtypes of sHLH.

A total of 49 patients (44.5%) received chemotherapy (HLH-1994/2004 regimen), with the treatment decision based on disease severity, the identified underlying etiology, and inadequate response to first-line immunosuppressive therapy. Among them, 24 received HLH-1994, 17 received HLH-2004, and 8 received plasma exchange (PE) combined with the HLH-1994/2004. In addition, 3 patients relapsed during continuation therapy of the HLH-1994 protocol and were started on salvage chemotherapy using the L-DEP regimen. The remaining 61 cases (55.5%) did not undergo chemotherapy, of which 7 received pulse steroid therapy, 7 were treated with PE combined with steroids, and 10 received only symptomatic treatment (e.g., anti-infection/antiviral therapy, IVIG, granulocyte colony-stimulating factor). As shown in **Figure 2**, the therapeutic approaches varied significantly among sHLH subtypes. EBV-HLH had the highest chemotherapy application rate (75.5%), with a remission rate of 91.9%. While MAS patients predominantly underwent high-dose methylprednisolone pulse therapy in combination with cyclosporine.

Notably, 15 of 110 children with sHLH died during hospitalization, yielding an overall mortality rate of 13.6%, with MODS as the primary cause of death (**Table 3**). Meanwhile, 80 cases (72.8%) achieved clinical remission, 3 cases (2.7%) with refractory/recurrent EBV-HLH were scheduled for hematopoietic stem cell transplantation (HSCT), and 12 cases (10.9%) were discharged at guardians’ request with subsequent loss to follow-up. Detailed data for deceased patients are shown in **Table 3**.

**Table 3:** Summary of 15 deceased children with sHLH.

Characteristic	Case (n)	Percentage (%)
Sex		
Male	12	80.0
Female	3	20.0
Median age (Years)	1.0 (0.4, 2.5)	–
Pathogenesis		
Epstein-Barr virus	4	26.6
Respiratory syncytial virus	1	6.7
*Streptococcus pneumoniae*	1	6.7
*Candida glabrata*	1	6.7
Multiple pathogens	5	33.3
Neuroblastoma	1	6.7
Unknown etiology	2	13.3
Treatment		
HLH-1994/2004	3	20.0
PE+HLH-1994/2004	2	13.3
Glucocorticoid +IVIG	7	46.7
PE+Glucocorticoid +IVIG	3	20.0
Death causes		
MODS	11	73.3
Respiratory failure	2	13.3
CNSI	1	6.7
Sepsis	1	6.7

PE, Plasma exchange; IVIG, Intravenous immunoglobulins; CNSI, Central nervous system involvement; MODS, Multiple organ dysfunction syndrome.

## Discussion

HLH is a rare disease with rapid progression and high lethality (15). In recent years, with the in-depth medical research and continuous improvement of diagnostic techniques, understanding of pediatric HLH has gradually deepened. However, due to the diversity and complexity of its clinical manifestations, early diagnosis and effective treatment remain challenging. Epidemiologically, although the exact data vary to some extent across different regions and studies, the overall situation cannot be ignored (1, 2). Currently, most large-scale epidemiological studies report the overall incidence of HLH, while precise incidence data for sHLH subgroups remain limited. A retrospective study from a tertiary academic hospital in Texas, USA, indicated that the prevalence of HLH among children is approximately 1 in 100,000, with a median diagnosis age of 1.8 years (16). Yao et al. (17) conducted the first large-scale epidemiological survey of HLH in China, reporting that the highest incidence occurred in Gansu Province (approximately 0.468 per 100,000), with an overall decreasing trend from inland to coastal and border regions. However, precise subtype−specific incidence data for pediatric sHLH remain lacking. Our single−center data delineate the clinical profile of pediatric sHLH in northern China, providing a foundation for future multicenter epidemiological studies.

In this study, the median age of 110 children was 2.67 years, with 52.7% were younger than 3 years. Mao et al. (18) reported a median age of 2.0 years among 175 pediatric HLH cases, with 54.3% younger than 3 years; Zhou et al. (19) reported a median age of 2.1 years in 227 cases, and more than 60% younger than 3 years. Collectively, these studies confirm that infancy and early childhood represent the peak incidence period for pediatric sHLH. This age distribution likely reflects the immature immune system of young children, which is more susceptible to immune dysregulation following stimuli such as infections.

Etiologically, infection was the most significant predisposing factor (78.2%), with EBV infection playing an absolutely dominant role (57.3%), consistent with previous reports from China (19) and other countries (20, 21). This highlights the critical role of EBV screening in the diagnostic workup of pediatric sHLH. Notably, the etiology remains unknown in 12.7% of cases, which is consistent with the 14.9% reported by Yao et al. (17) in a multi-center study, indicating potential limitations of current diagnostic techniques or the presence of complex, concealed predisposing factors. Furthermore, 11.8% of IAHLH patients had multiple pathogen co-infections, which may be associated with the child’s immune status, infection routes, and pathogen interactions. Co-infections may act synergistically, enhancing immune system stimulation, further exacerbating immune disorders, and thereby increasing the risk and the complexity of sHLH, posing greater challenges to diagnosis and treatment, requiring greater attention from clinicians (7, 22, 23). However, autoimmune and tumor-related HLH were relatively rare in our cohort, which might be related to regional differences and age distribution.

The clinical manifestations of sHLH in our cohort fully reflect the systemic and multi-organ involvement characteristics, with fever, lymphadenopathy, splenomegaly, and hepatomegaly as the most common manifestations, aligning with the classic HLH phenotype described in the literature (19, 24), In addition, a critical finding of our study was the high prevalence of respiratory system involvement: 58.2% of patients were diagnosed with pneumonia by imaging, 36.4% had pleural effusion, and 21.8% developed respiratory failure requiring mechanical ventilation. A similarly high rate of respiratory involvement was also reported by Luo et al. (25), with pneumonia in 75% and respiratory failure in 41.4% of pediatric sHLH patients. The pulmonary inflammatory may not only from secondary infections but also from the continuous activation of mononuclear macrophages, which secrete a large amount of pro-inflammatory factors such as tumor necrosis factor α (TNF-α), IL-1β, and IL-6 (26, 27). These factors infiltrate the lungs, leading to pulmonary inflammation and edema, interfering with gas exchange, and resulting in symptoms such as cough, expectoration, wheezing, and dyspnea. Severe pulmonary lesions can cause respiratory failure, posing a threat to the lives of children (28, 29). This finding highlights that respiratory system involvement is an important clinical feature of pediatric sHLH.

The reported incidence of CNSI in sHLH patients varies significantly across studies (24, 30, 31), which may be attributed to differing diagnostic criteria among researchers. In our cohort, CNSI occurred in 22.7% of cases. Although some cases of convulsions and lethargy could not be ruled out as secondary to persistent high fever, children with lethargy and convulsions in this study often also exhibited imaging abnormalities, and a small number had abnormal cerebrospinal fluid findings. Increasing evidence suggests that CNSI serves as an independent risk factor for poor prognosis in HLH (32, 33). Historically, pediatrician may have overlooked manifestations of CNSI, as the majority of pediatric patients present with subtle or absent symptoms at disease onset, with clinical manifestations becoming apparent only during the disease course. Consequently, comprehensive neuroimaging and cerebrospinal fluid analysis are paramount for the diagnosis of CNS-HLH (8).

Laboratory test results further confirm the pathophysiological process of sHLH. Characteristic laboratory abnormalities such as pancytopenia (especially thrombocytopenia), hyperferritinemia, and hypofibrinogenemia have a high incidence in this group of children. This is highly consistent with the indicators in the HLH-2004 diagnostic criteria (12). The diagnostic value of elevated serum ferritin in pediatric and adult patients with HLH has consistently attracted significant attention. Hyperferritinemia was observed in 89.1% of our patients, slightly higher than the 85.1% and 85.0% reported by Mao et al. (18) and Zhou et al. (19), Respectively. Giemza-Stokłosa et al. (34) described that excessive ferritin can induce cytotoxic damage to histiocytes, exacerbate organ injury, and is closely linked to the severity and prognosis of sHLH.

Liver damage was particularly prominent in this group of sHLH patients. As noted, two-thirds of the children had hepatomegaly (66.4%), and most had elevated liver enzymes, especially LDH (84.6%) and AST (82.7%). ALT and HBDH were also elevated to varying degrees, with frequencies of 70.9% and 65.5%, respectively. Our findings align with previous reports, for instance, Oguz et al. (21) reported elevated LDH was a universal finding (100%), and AST and ALT were elevated in 87.5% of their cohort. Further, 30.9% of the patients showed hypoalbuminemia, and 50% of patients developed hypofibrinogenemia. The degree of liver function impairment reflects the extent of immune dysfunction and inflammatory damage in sHLH (35). Severe liver impairment leads to impaired synthesis of albumin, fibrinogen, and corresponding coagulation factors, which, combined with the “superimposed effect” of thrombocytopenia caused by hemophagocytic activity, may result in severe bleeding and even death in pediatric sHLH patients (3). Therefore, liver function-related indicators are of great auxiliary value for diagnosing sHLH.

HLH as a clinically heterogeneous syndrome with diverse etiologies, however, all cases manifest clinical symptoms and laboratory abnormalities stemming from immune dysregulation, hyperactivation, and immune-mediated tissue damage (36). Consequently, immunological markers hold significant diagnostic value for HLH. According to the HLH-2004 diagnostic criteria, elevated sCD25 levels and reduced NK cell activity constitute essential diagnostic parameters. Nevertheless, the widespread implementation of these tests in routine clinical practice is constrained by their technical complexity, sophisticated equipment requirements, and substantial costs. This study evaluates immunological status changes in pediatric patients by analyzing the distribution of peripheral blood lymphocyte subsets. The most frequent alterations included a decreased NK cell rate (75.5%). Concurrently, we observed T-cell compartment skewing, evidenced by an elevated CD3^+^ cell rate (69.1%) driven primarily by an increase in CD8^+^ T cells (60.9%), which resulted in a decreased CD4^+^/CD8^+^ ratio (59.1%). Abnormal lymphocyte subsets in pediatric HLH have been reported in smaller cohorts, such as the study by An et al. (37), but large-sample data remain scarce. Our study helps to address this gap and further demonstrates that sHLH is characterized by pronounced T−cell activation and immune imbalance. The analysis of these readily available peripheral blood lymphocyte subsets, which can serve as a strong auxiliary clue to support the diagnosis of sHLH.

Notably, cases are widely distributed across 12 departments, including the hematology department, PICU, and rheumatology and immunology department. This fully reflects the high complexity and diversity of the clinical manifestations of pediatric HLH. Since the symptoms of HLH are similar to those of multiple systemic diseases, it is prone to misdiagnosis and missed diagnosis. This characteristic of multidepartment distribution not only increases the difficulty for clinicians in diagnosing HLH, but also alerts clinicians that when children present with persistent fever, hepatosplenomegaly and lymphadenopathy, poor anti - infection efficacy, as well as abnormal liver function and cytopenia, they should be vigilant for HLH.

Therapeutically, our data highlight the importance of individualized strategies. Nearly half of the patients received the HLH-94/04 chemotherapy protocol, among them, three-quarters children had EBV infection, and the chemotherapy remission rate reached 91.9%. A study by Japanese scholars also indicated that the HLH -2004 regimen has a significant therapeutic effect on children with EBV-related HLH (38). In contrast, MAS patients responded well to pulse steroid therapy combined with cyclosporine, with no in-hospital deaths, which further supports the necessity of subtype-specific treatment approaches for sHLH. Although HLH-94/04-based chemo-immunotherapy can achieve initial remission in some HLH children (39, 40), allogeneic hematopoietic stem cell transplantation (allo-HSCT) remains the curative treatment for primary HLH and the core salvage therapeutic strategy for relapsed/refractory HLH (41). In our cohort, 3 children with relapsed/refractory EBV-HLH underwent allo-HSCT after salvage chemotherapy, which confirms that timely initiation of allo-HSCT intervention is a key salvage strategy to improve prognosis for patients with poor response to conventional chemo-immunotherapy. Recently, targeted biologic agents have also shown promising results. Ruxolitinib (a JAK1/2 inhibitor) and emapalumab (an anti-IFN-γ monoclonal antibody) have shown promising results in the treatment of refractory/relapsed HLH by mitigating the cytokine storm (42–45). Anakinra (an IL-1 receptor antagonist) has also been shown to rapidly control hyper-inflammation in MAS and infection-associated HLH, with good tolerability in pediatric populations (46, 47). These agents offer novel mechanisms of action and can serve as bridges to HSCT or components of combination therapy.

The overall in-hospital mortality rate of 13.6% was lower than previously reported rates (21.6%-37.1%) (18, 33, 48), likely attributed to early diagnosis (median diagnostic time: 3 days) and timely implementation of individualized intervention strategies. A comprehensive study spanning nearly two decades (2001-2018) also demonstrated that most deaths occur in the early stage of the disease, and mortality has significantly decreased to 16.4% in the recent six years, suggesting improved clinical awareness of this fatal pediatric disease may contribute to reduced mortality (19). Our research shows that MODS was the leading cause of death, further indicating that sHLH often presents as a fulminant, multi-systems disorder. Thus close monitoring of organ function and timely intervention for organ injury are critical for high-risk sHLH patients.

This study also has certain limitations. Its single-center design may not fully reflect all clinical features and patterns of pediatric HLH. The retrospective research method has inherent limitations, without follow - up data tracking, issues such as information omission and bias may occur. Future multicenter collaborative research with long-term follow-up is needed to enhance the representativeness and reliability of findings. At the same time, research on disease risk factors should be strengthened to identify high-risk groups early and implement effective preventive measures.

In summary, sHLH is a heterogeneous disorder with diverse etiologies and clinical manifestations, primarily affecting infants and young children, among whom EBV infection is a key pathogenic factor. The HLH-2004 diagnostic criteria have a solid theoretical basis and practical clinical value. The frequent occurrence of hepatitis-related damage indicators, abnormalities in peripheral blood lymphocyte subsets and respiratory system involvement highlights their potential utility as auxiliary clues for raising clinical suspicion and supporting the diagnostic process. In addition, our study found that treatment efficacy varies among sHLH subtypes, necessitating corresponding tailored strategies. For suspected sHLH children, early diagnosis and timely treatment are key measures to improve prognosis.

## Data Availability

The original contributions presented in the study are included in the article/supplementary material. Further inquiries can be directed to the corresponding authors.
